# Bringing Untargeted Metabolomics to the Clinic: Real‐World Data and Implementation for Screening of Inherited Metabolic Diseases

**DOI:** 10.1002/jimd.70260

**Published:** 2026-11-09

**Authors:** D. Augustijn, R. Bonte, M. C. Hagemeijer, J. Bierau, E. H. Jacobs, C. J. Klein‐de Ronde, E. van der Star, D. Y. van Haaften‐Visser, N. C. Peltenburg‐de Jong, C. Debuy, M. van Dijk, D. Vos, G. J. G. Ruijter, S. M. I. Goorden

**Affiliations:** ^1^ Centre of Lysosomal and Metabolic Diseases, Department of Clinical Genetics Erasmus Medical Centre Rotterdam the Netherlands; ^2^ Department of Clinical Chemistry Erasmus MC Rotterdam the Netherlands; ^3^ Centre of Lysosomal and Metabolic Diseases, Department of Paediatrics Erasmus Medical Centre Rotterdam the Netherlands; ^4^ Centre of Lysosomal and Metabolic Diseases, Department of Internal Medicine Erasmus Medical Centre Rotterdam the Netherlands; ^5^ Laboratory Genetic Metabolic Diseases, Department of Laboratory Medicine Amsterdam University Medical Centre Amsterdam the Netherlands

**Keywords:** diagnostics, IMD, implementation, metabolomics

## Abstract

Inherited metabolic diseases (IMD) require timely diagnosis since a considerable number of these disorders respond to early treatment. Traditional metabolic screening relies on multiple targeted assays guided by clinical symptoms, making it laborious and potentially increasing the risk of underdiagnosis. Untargeted metabolomics provides broader biochemical coverage, but evidence of its diagnostic performance is limited. In this metabolomics implementation study, we analysed 484 plasma samples submitted for routine metabolic screening using both targeted assays and metabolomics in parallel. The results of these analyses were independently interpreted by two laboratory specialists and results were compared. Overall, 85.5% concordance was achieved between the two approaches. The majority of discrepancies stem from aberrant biomarkers traditionally measured in urine observed in metabolomics but not in the targeted plasma analyses. Importantly, inconclusive results requiring follow up were equally observed among the two methods. Metabolomics screening resulted in the detection of one new and one tentative IMD, which were not detected by targeted tests. Collectively, these results support the application of untargeted metabolomics for IMD screening in clinical practice. In addition to our study results, we share our detailed protocol and workflow towards implementation of untargeted metabolomics in clinical practice to facilitate broader use of diagnostic metabolomics in the IMD community.

**Abbreviations**
HPO: Human Phenotype Ontology
IMD: inherited metabolic diseases
LC: liquid chromatography
MS: mass spectrometry
OTC: ornithine carbamoyltransferase
QC: quality control
TMLHE: epsilon‐trimethyllysine hydroxylase
UHPLC‐HRAM‐MS/MS: ultra high‐performance liquid chromatography high‐resolution accurate mass spectrometry
VUS: variants of unknown significance

## 1 Introduction

Inherited metabolic diseases (IMD) are rare genetic disorders caused by deficiencies of specific enzymes and transporters that affect key metabolic processes and result in buildup of toxic intermediates and/or deficiencies of metabolic end‐products. They typically manifest early in life, but late‐onset presentations are increasingly recognised as well, thereby expanding the clinical spectrum. Early diagnosis is essential since a considerable number of these disorders respond to timely treatment.

Traditionally, the screening for IMD, referred to as selective metabolic screening, involves the application of multiple targeted assays, depending on clinical symptoms, that measure specific groups of metabolites in plasma and urine. This is a laborious process and poses the risk of underdiagnosis, especially when clinical symptoms are incompletely provided or clinical presentation is atypical and can additionally be limited by the availability of targeted tests.

Untargeted metabolomics is an emerging approach in the laboratory diagnosis of IMD (reviewed in [1]). Despite its relative novelty, however, it is the most prevalent clinical application of metabolomics, according to a recent survey of the IFCC Metabolomics Working Group [2]. This technique allows the simultaneous (semi‐)quantification of hundreds to thousands of metabolites belonging to different classes of compounds, circumventing the pitfalls of selective metabolic screening as mentioned above.

Importantly, there are significant differences between metabolomics approaches and selective metabolic screening that should be considered when applied in clinical practice. Targeted quantitative tests are fully optimised for a specific panel of metabolites to allow comparison with established reference intervals. In contrast, metabolomics uses a general extraction method and a limited set of internal standards, resulting in semi‐quantitative results. Here, results are reported as *z*‐scores or fold‐changes compared to control samples and require a pattern recognition approach for interpretation. While metabolomics has proven to be sensitive in diagnosing different IMD in a validation setting [3, 4, 5, 6, 7, 8], published real‐world experience is scarce [9, 10, 11].

As such, we tested the performance of our validated metabolomics platform [6] in clinical practice in this metabolomics implementation study. Plasma samples sent in for routine metabolic screening were included and analysed in parallel using both targeted tests and metabolomics. Additionally, for patients that were diagnosed with an IMD in the timeframe of the study, but did not meet the inclusion criterion, we performed plasma metabolomics afterwards. We studied the concordance between targeted tests and metabolomics and assessed if targeted plasma amino acid and acylcarnitine analyses could safely be replaced by metabolomics for IMD screening. Furthermore, our broader objective was to share and discuss our detailed protocol, including internal and external QC procedures and workflow and the road to implementation of metabolomics in clinical practice.

## 2 Materials and Methods

### 2.1 Sample Inclusion

We aimed to include 500 patients referred for routine metabolic screening and accordingly set the study period to 7 months. Samples sent in for therapy monitoring, positive neonatal screening follow‐up, functional characterisation of genetic variants of unknown significance (VUS) and emergency requests were excluded. Targeted tests and metabolomics experiments were performed on the same heparinised plasma samples from each patient. This study was conducted in accordance with local legislation and institutional guidelines. All patients or their legal guardians approved the use of their remnant samples from routine care for method development and validation, in agreement with institutional and national legislation.

### 2.2 Metabolomics

Metabolomics was performed as described previously in [6] using ultra high‐performance liquid chromatography high‐resolution accurate mass spectrometry [UHPLC‐HRAM‐MS(/MS)] analysis (visualised in Figure 1). Semi‐automated sample preparation was performed on a Hamilton Robotics ML‐STAR eight channel liquid‐handling station (Hamilton, Bonaduz, Switzerland) using Phree 96‐well plates (Phenomenex, Torrance, CA, USA). Sample preparation and measurements were performed in triplicate using 75 μL of heparinised plasma, although a minimum volume of 120 μL was required for the pipetting robot. One run allowed the extraction and analysis of 24 patient samples, two controls, a pooled sample and a blank.

**FIGURE 1:**
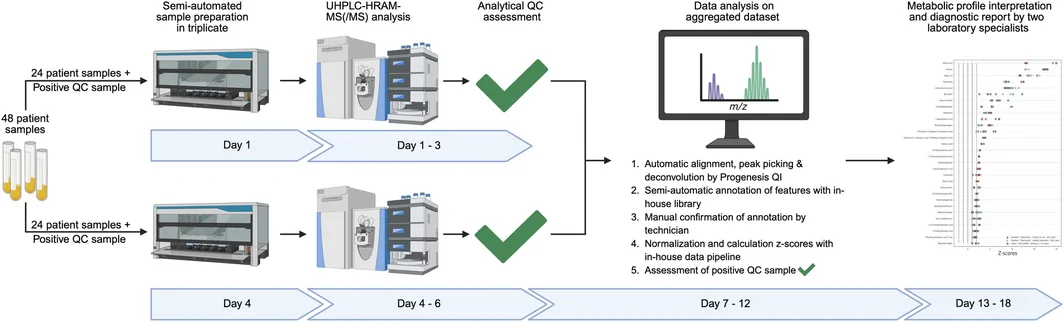
Visualisation of the applied metabolomics protocol. QC = quality control; UHPLC‐HRAM‐MS(/MS) = ultra high‐performance liquid chromatography high‐resolution accurate mass spectrometry. Created in https://BioRender.com.

UHPLC‐HRAM‐MS(/MS) analysis was performed using a Dionex Ultimate 3000 UHPLC chromatographic system combined with a Q Exactive Plus mass spectrometer (Thermo Fisher Scientific, Breda, the Netherlands). Xcalibur 4.3.73.11, SII 1.5 and MSTune 2.11 QF 1 (Thermo Fisher Scientific) were used as software interfaces. UHPLC separation was performed on a Kinetex F5 2.6 μm 2.1 mm × 150 mm column equipped with an F5 guard column (Phenomenex). Data analysis was performed on a set of 48 patient samples (two experimental runs) using aggregated analysis. The raw data were imported into Progenesis QI v3.0 (Waters, Newcastle upon Tyne, UK), which allows alignment of the chromatograms, normalisation, deisotoping, adduct deconvolution, peak picking and peak annotation.

Compound ions were provisionally annotated by matching the retention time (max. ΔRT: 0.15 min), isotope pattern similarity (> 85%) and m/z‐value of a feature (max. Δ ppm: 3) with an in‐house developed database. This database encompassed 879 metabolites, of which 540 metabolites had an established retention time. Annotation was manually confirmed by a well‐trained technician. Annotation levels of specific metabolites could differ between experimental runs, depending on their abundance in the measured samples.

After these processing steps, a matrix of abundances was obtained for the Progenesis QI experiment, with rows corresponding to features and columns to samples or metadata (e.g., feature id, retention time, max fold change or annotation). These measurement results were exported as CSV files and processed using an in‐house data pipeline that normalised the data by Box–Cox probabilistic quotient normalisation. Prior to *z*‐score calculation, metabolites with outlying values were excluded as described in [12]. For each metabolite, within‐batch *z*‐scores were calculated for each triplicate, using the other 47 patient samples (from the 2 experimental runs) as reference population.

Between‐batch *z*‐scores were computed by integrating data from multiple batches after normalisation with the Metchalizer method [12]. Between‐batch *z*‐scores were only calculated for metabolites annotated in at least seven batches, either with or without the application of a regression model correcting for sex and age. Thus, the resulting output per metabolite consisted of three uncorrected and three corrected between‐batch *z*‐scores.

The bioinformatics pipeline employed in this study is available through GitHub (https://github.com/mbongaerts/Metchalizer). For further details necessary for its correct implementation, readers are encouraged to contact the corresponding author.

### 2.3 Quality Control (QC)

Each experimental run included two types of quality control samples (referred to as analytical‐ and positive QC). For the analytical QC, two sets of isotopically labelled internal standards were used. A set of nine internal standards was added prior to protein precipitation to monitor total analytical variation. Another set of six isotopically labelled standards—referred to as external standards to distinguish them from the first set—was added in the final step of sample preparation to specifically monitor variation arising during the measurement of processed samples. Second, a positive QC sample was included to assess the analytical performance of the entire workflow, including sample preparation, measurement and data analysis. This QC consisted of urine samples from patients with seven different IMD: argininosuccinate lyase deficiency (OMIM # 207900), multiple acyl‐CoA dehydrogenase deficiency (OMIM # 231680), thymidine phosphorylase deficiency (OMIM # 131222), HMG‐CoA lyase deficiency (OMIM # 246450), aspartylglucosaminuria (OMIM # 208400), cerebrotendinous xanthomatosis (OMIM # 213700) and cobalamin C deficiency (OMIM # 277400).

Prior to analysis of the experimental runs in Progenesis QI, raw data quality was assessed. For this purpose, internal and external standards in all plasma samples were integrated using Xcalibur and manually reviewed in the Quan Browser module.

[D5]‐phenylalanine, 1,3‐[15N]‐uracil, [D10]‐isoleucine, [D6]‐ornithine, [D4]‐tyrosine, 5‐bromo‐DL‐tryptophan, 3,3‐dimethylglutaric acid, [D3]‐carnitine, [D2]‐uridine, [D8]‐valine, [D2]‐acetylcarnitine, [D3]‐hexanoylcarnitine, [D3]‐tetradecanoylcarnitine and [D3]‐hexadecanoylcarnitine were monitored in positive ion mode whereas [D5]‐phenylalanine, [13C]‐thymidine, 1,3‐[15 N]‐uracil, [D4]‐tyrosine, 5‐bromo‐DL‐tryptophan, 3,3‐dimethylglutaric acid, [D4]‐glycochenodeoxycholic acid, [D3]‐methylmalonic acid, [D2]‐uridine and [D8]‐valine were monitored in the negative ion mode. Next, two separate Long Excel reports (one for each ion mode) were generated using the *export Data to Excel* function. Subsequently, these files were processed by an in‐house QC pipeline to automatically extract QC data.

For every experimental run, a report was generated containing intra‐ and inter‐batch CVs of the peak areas and retention times of all standards, together with absolute peak areas and retention times over time to allow trend analysis.

An experimental run had to meet the following QC criteria for all standards:

- Within batch CV of peak area < 30%.
- Within batch CV of retention time < 1%.
- Absolute average peak area per batch < ±3 SD of reference peak area.
- Between batch *z*‐score of |intra batch‐CV| of peak area < 3.
- Between batch *z*‐score of |intra‐batch CV| on retention time < 3.

The results of the positive control were evaluated after data processing in Progenesis QI and the in‐house data analysis pipeline. The analytical performance of the entire workflow was considered satisfactory when all metabolites detected in the positive control sample demonstrated *z* > 3. This included nine metabolites measured in negative ion mode, thirteen metabolites in positive ion mode and one metabolite detected in both ionisation modes.

### 2.4 External Quality Assessment for Diagnostic Metabolomics

Proficiency of diagnostic metabolomics was assessed by participation in a Dutch sample exchange programme in which five centres participated. We scored 88% proficiency during the study period (*n =* 8). One diagnosis of a patient with non‐ketotic hyperglycinemia (NKH) was missed. Notably, only 2 out of the 5 participating laboratories correctly diagnosed this sample.

### 2.5 Selective Metabolic Screening

Targeted analyses (except for carnitine biosynthesis intermediates) were performed according to ISO15189 guidelines using established methods. Amino acid analysis was performed by ion exchange chromatography, acylcarnitine analysis by flow injection tandem mass spectrometry (MS), sialotransferrin analysis by isoelectric focusing, methylmalonic acid and total homocysteine by liquid chromatography (LC)–MS/MS and very long‐chain fatty acids analysis by gas chromatography–MS. Carnitine biosynthesis intermediates and pipecolic acid were measured using LC–MS/MS at the laboratory for Genetic Metabolic Diseases (laboratory GMD) at Amsterdam UMC, the Netherlands.

### 2.6 Conversion of Local Clinical Codes Into HPO Terms

We manually converted the symptoms reported by referring physicians according to a local coding system into the corresponding Human Phenotype Ontology (HPO) terms [13] (according to January 2026 website version). For the subgroup analysis depicted in Figure 4D individual HPO terms were clustered according to their higher‐level parent term in the HPO hierarchy.

### 2.7 Statistical Analysis

Statistical analysis was performed using R 4.5.2 including the descriptive statistics of the cohort and the concordance analysis package. The Chi‐square or Fisher's exact test was used to test for significant differences between categorical variables. The correlation between quantitative concentrations of the targeted tests and metabolomics *z*‐scores was assessed using Pearson's correlation coefficient.

## 3 Results

### 3.1 Study and Cohort Description

During the study period 492 samples were received for IMD screening, of which 484 samples had approved (parental) consent (see Figure 2). In these samples, a selection of targeted analyses based on clinical indication was performed with subsequent reporting by a diagnostic letter according to routine diagnostic workup. In parallel, metabolomics was performed, independently interpreted and scored by two certified laboratory specialists, who were uninformed about the outcomes of the targeted tests.

**FIGURE 2:**
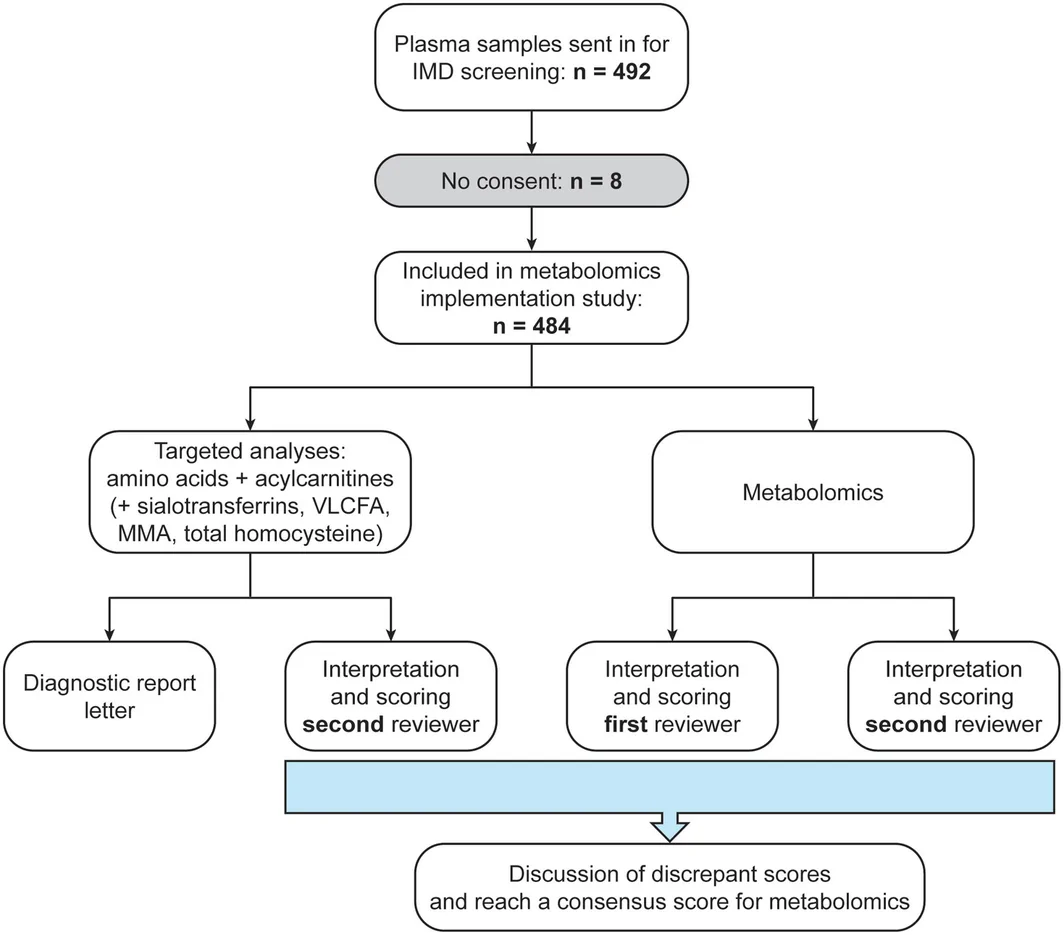
Diagram depicting the study design. IMD = inherited metabolic disorder; MMA = methylmalonic acid; VLCFA = very long chain fatty acids.

The majority of the patients were children (84.1%, median age 5.1 years), mostly external referrals (64.3%) (see Table 1). Most referrals originated from the paediatrics departments, followed by the neurology and clinical genetics departments. Notably, a bias toward the male sex was observed (62.0%). In 80.0% of the cases, we also received a urine sample, which is consistent with our diagnostic guidelines. In line with standard practice in selective metabolic screening, amino acids and acylcarnitines were analysed in all samples, while sialotransferrins were analysed in 98.1% of cases. Additional analyses (very long‐chain fatty acids and phytanic and pristanic acid, total homocysteine and methylmalonic acid) were included in selective metabolic screening guided by the clinical context.

**TABLE 1:** Patient and sample characteristics.

Number of patients	484
Median age in years (range)	5.1 (2 days to 75.4 years)
Number of children (%)	407 (84.1%)
Neonate (< 28 days)	14
Infant (28 days to 1 year)	35
Young child (1–4 years)	187
Older child (5–12 years)	127
Adolescent (12–18 years)	44
Median age children in years (range)	4.4 (2 days to 17.9 years)
Number of adults (%)	77 (15.9%)
Median age adults in years (range)	37.4 (18.6–75.4)
Sex (% female)	184 (38.0%)
Number of external referrals (%)	311 (64.3%)
Medical discipline (%)	
Paediatrics	291 (60.1%)
Neurology	58 (12.0%)
Clinical Genetics	50 (10.3%)
Paediatric Neurology	42 (8.7%)
Internal Medicine	22 (4.5%)
Intellectual Disability Medicine	20 (4.1%)
Psychiatry	1 (0.2%)
Urine samples received (%)	387 (80.0%)
Diagnostics test (%)	
Amino acids	484 (100%)
Acylcarnitines	484 (100%)
Sialotransferrins	475 (98.1%)
Methylmalonic acid	4 (0.8%)
Homocysteine	64 (13.2%)
Very long‐chain fatty acids	87 (18.0%)

The most frequently reported clinical presentations were intellectual disability (46.7%), autism (23.1%) and motor delay (22.5%) (see Figure 3). Because of the imbalance between male and female patients in our cohort, we analysed the prevalence of symptoms stratified by sex (see Supplementary Figure 1). Intellectual disability (53.3% vs. 35.9%, *p* < 0.001) and autism (29.0% vs. 13.6%, *p* < 0.001) were more frequently reported in male patients compared to female patients. The remaining clinical symptoms were equally distributed between sexes.

**FIGURE 3:**
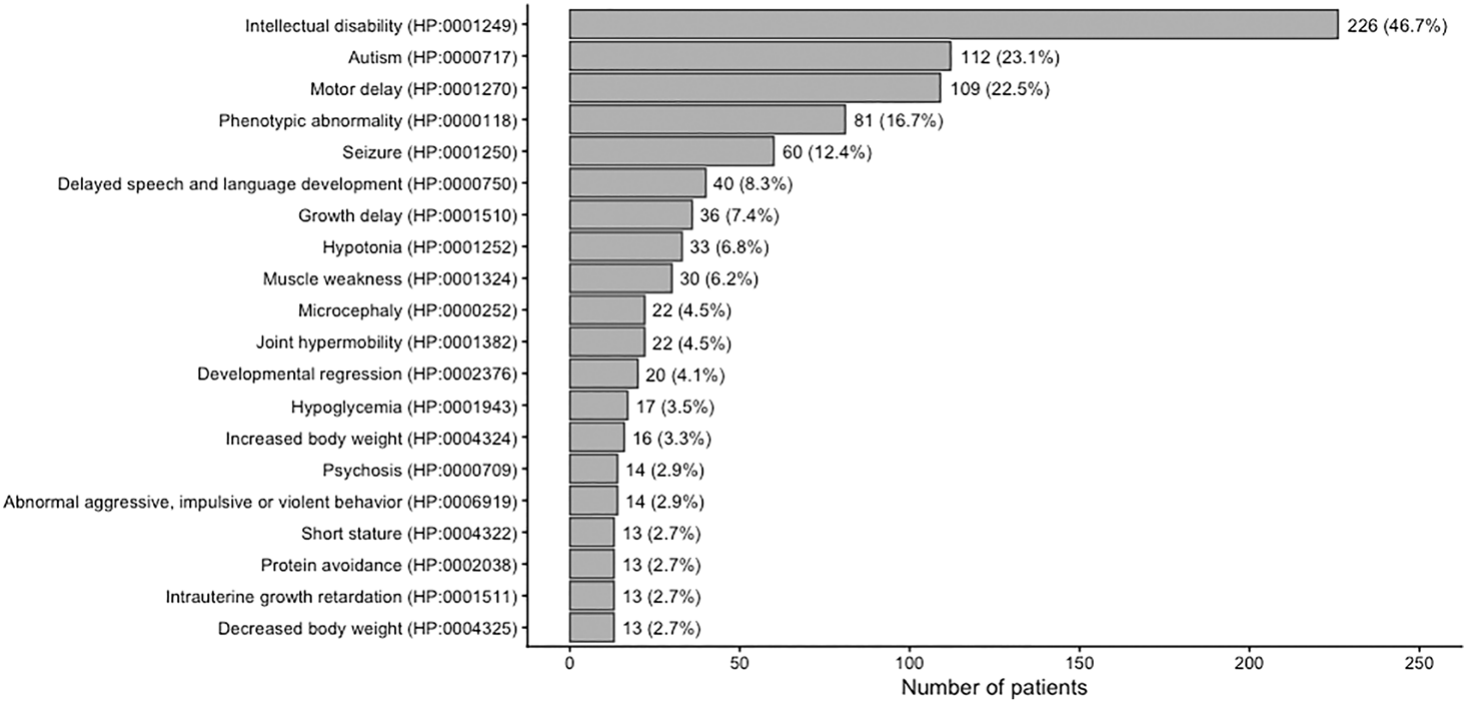
Most frequent clinical indications in the study population according to HPO terminology.

### 3.2 Comparison of Selective Metabolic Screening and Metabolomics Results

To reach concordant results between the two analysis techniques, it is a prerequisite that targeted concentrations and *z*‐scores show considerable correlation. We tested this for a selection of diagnostically relevant amino acids and acylcarnitines (see Supplementary Figure 2). Using Pearson correlation analysis, a significant positive correlation (*p* < 0.01) was observed between quantitative assays and metabolomics *z*‐scores. For most metabolites, the Pearson correlation coefficient (*r*) is > 0.7, indicating a strong association [14].

Metabolomics analyses were reported as four *z*‐score plots: two ranking the most elevated metabolites in positive and negative ionisation mode and two with the most decreased metabolites (an example *z*‐score plot is depicted in Supplementary Figure 3). These plots were independently interpreted by two laboratory specialists using the scoring system depicted in Figure 4A. This scoring system allowed for direct comparison between the results of the amino acids and acylcarnitines with (un)targeted analyses in plasma (scores 1–3) but also exploration of possible additional biomarkers for IMD using other assays and/or matrices (scores A–B). Two types of discrepancies were observed between (i) the targeted assays and metabolomics results and (ii) the interpretation of two laboratory specialists. Both were discussed in 16 1‐h sessions, involving all five laboratory specialists, to reach a metabolomics consensus score for further analysis.

**FIGURE 4:**
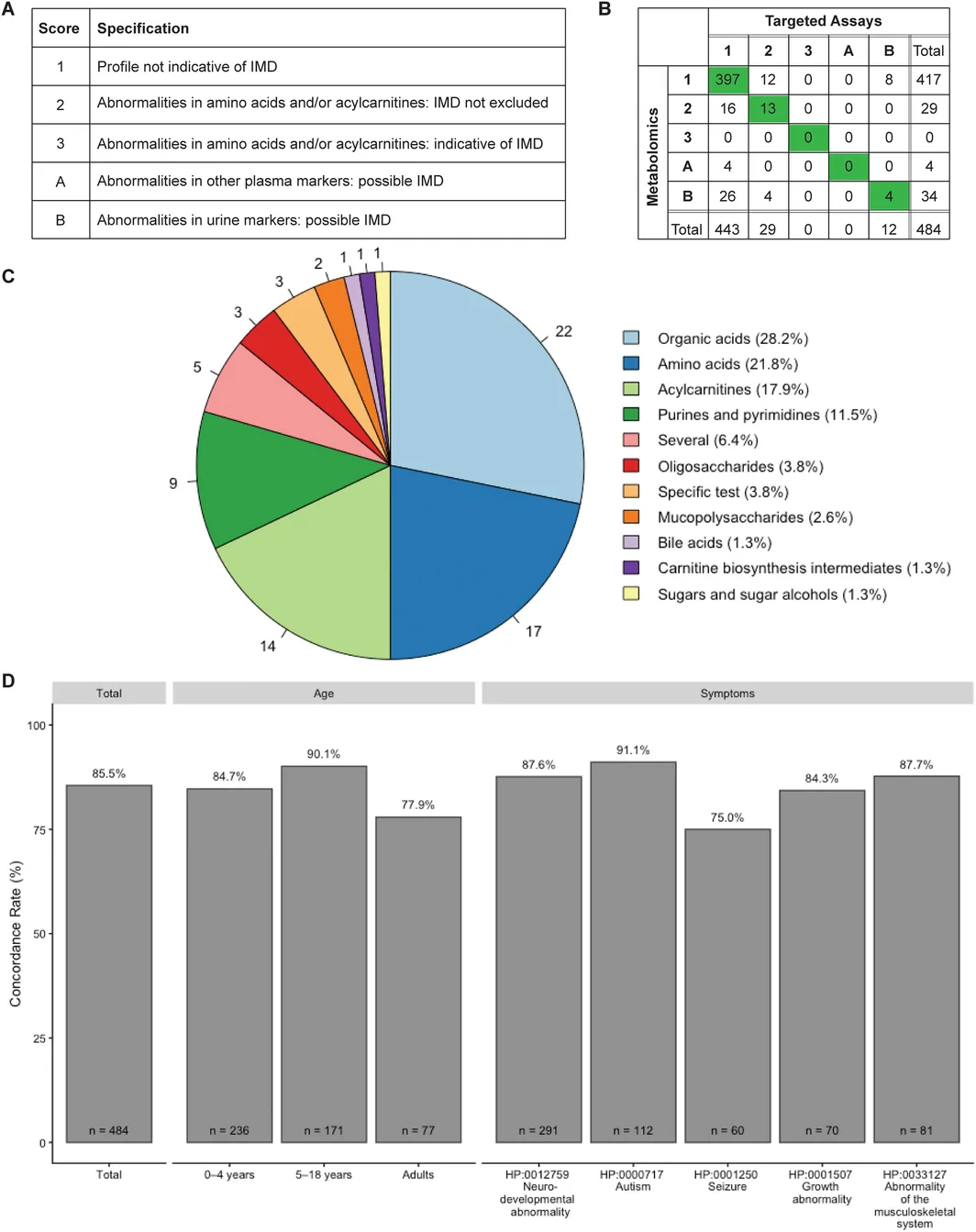
Concordance between targeted tests and metabolomics. (A) Scoring system applied to the results of targeted tests and metabolomics. Urine markers: metabolites which are traditionally measured in urine by targeted tests. (B) Concordance table showing the agreement between results of the two analysis methods using the scoring system depicted in A. (C) Attribution of each metabolite group to the discordance between metabolomics and targeted tests. (D) Concordance stratified by age and symptoms. Patients may present with multiple features and may therefore be included in different subgroups.

As can be seen in Figure 4B, the agreement between the targeted tests and metabolomics was 85.5% (95% CI: 82.1%–88.5%; *n* = 414). Most of the discordance could be attributed to abnormalities in urine biomarkers (score B) that were observed in metabolomics but not in the targeted analyses. Most of these discrepancies concerned elevated plasma organic acids levels in metabolomics (*n* = 22), which are traditionally measured in urine, followed by scoring differences in amino acids (*n* = 17) and acylcarnitines (*n* = 14) (see Figure 4C). Importantly, score 2 results (IMD not excluded and requiring extra investigations, e.g., measurement of lactate or ammonia) or repeated targeted measurement(s) were equally frequent in both methods (6.0%), indicating that applying metabolomics does not result in additional follow up investigations. Detailed information about the samples that showed discrepancies in scoring between the quantitative tests and metabolomic analyses can be found in Supplementary Table 1.

Next, we performed a post hoc subgroup analysis to test whether metabolomics performed equally well in different groups stratified by age and clinical presentation based on clustered HPO terms (see Figure 4D). We found no significant difference between the agreement reached in different age groups or clinical presentations, including the seizure cluster which showed the lowest concordance rate (75% vs. overall 85.5%; *p* = 0.058).

Within the study, no IMD were diagnosed based on the results of targeted plasma tests, while in the study timeframe we diagnosed four new patients using targeted urine tests (P‐92, P‐251, P‐442 and P‐474, see Table 2). The metabolic profile in plasma of these four IMD patients was scored as normal (score = 1). Notably, we found one additional new diagnosis (P‐334) and one tentative diagnosis (P‐436) based on the metabolomic screening results, corresponding to an additional diagnostic yield of 0.2% if P‐436 is excluded and 0.4% if included using metabolomics.

**TABLE 2:** Summary of all IMD diagnoses established within the study period with associated scores and abnormal metabolites.

Sample	Score metabolomics	Score targeted tests	Aberrant metabolites metabolomics related to diagnosis	Aberrant metabolites targeted tests related to diagnosis	Diagnosis (# OMIM number)
IMD diagnoses established within the pilot, based on targeted urine tests					
P‐93	1	B	None	*Elevated excretion* – Tiglylglycine (9 mmol/mol creatinine, RI = 0–5) – 2‐methyl‐3‐hydroxybutyric acid (qualitative, RI = n.d.)	HSD17B10 deficiency (#300438)
P‐351	1	B	None	*Elevated excretion* – *Total mucopolysacharides* (8.2 mmol/mol creatinine, RI = 0–6.7) – *Heparan sulphate* (6.9 mmol/mol creatinine, RI = 0–1.8)	Mucopolysaccharidosis type IIIB: (#252920)
P‐442	1	B	None	*Elevated excretion* – *Total mucopolysacharides* (17.4 mmol/mol creatinine, RI = 0–11.2) – *Heparan sulphate* (18.3 mmol/mol creatinine, RI = 0–1.8) – *Dermatan sulphate* (10.6 mmol/mmol creatinine, RI = 0–3.0)	Mucopolysaccharidosis type IH (#607014)
P‐474	1	B	None	*Elevated excretion* – *Phosphoethanolamine* (258 mmol/mol creatinine, RI = 0–40).	Infantile Hypophosphatasia (#241500)
(Tentative) IMD diagnoses established within the pilot, based on metabolomics					
P‐334	B	1	*Elevated* – *Orotic acid* (*z*‐score = 9.0) *Decreased* – *Citrulline* (*z*‐score = −2.4)	*Decreased concentration* – *Citrulline* (7.7 μmol/L, RI = 16–56)	Ornithine Carbamoyltransferase deficiency (#311250)
P‐436	A	1	*Elevated* – *Trimethyllysine* (*z*‐score = 9.3) *Decreased* – *Deoxycarnitine (=gamma‐butyrobetaine)* (*z*‐score = −4.5).	*Elevated* – *Trimethyllysine* ^a^ (2.5 μmol/L, RI = 0.2–1.2) *Decreased* – *Deoxycarnitine (=gamma‐butyrobetaine)* ^a^ (0.06 μmol/L, RI = 0.3–1.4)	*Tentative*: Epsilon‐trimethyllysine hydroxylase deficiency (#300872)

Sample	Score metabolomics	Score targeted tests	Aberrant metabolites metabolomics related to diagnoses	Aberrant metabolites targeted tests related to diagnosis	Diagnosis (# OMIM number)	Indication for metabolic diagnostics
IMD diagnoses established outside the pilot, but within the study period						
E‐001	B	B	*Elevated* – *S‐sulphocysteine* (*z*‐score = 8.4) – *Xanthine* (*z*‐score = 13.4) – *Xanthosine* (*z*‐score = 42.7) *Decreased* – *N‐Methyl‐2‐pyridone‐5‐carboxamide* (*z*‐score = −4.0) – *N‐Methyl‐4‐pyridone‐5‐carboxamide* (*z*‐score = −3.1)	*Elevated excretion* – *S‐sulphocysteine* (131.9 mmol/mol creatinine, RI = 0.2–8.4) – *Alpha‐aminoadipine semialdehyde* (5.5 mmol/mol creatinine, RI = 0–2.4). – *Hypoxanthine* (287 mmol/mol creatinine, RI = 1–49) – *Xanthine* (667 mmol/mol creatinine, RI = 3–42)	Molybdenum cofactor deficiency (#252150)	Patient on the ICU with epilepsy
E‐002	3	3	*Elevated* – *Methylmalonic acid* (*z*‐score = 49.0) – *C4DC | methylmalonylcarnitine* (*z*‐score = 19.9) – *C3 | propionylcarnitine* (*z*‐score = 4.2)	*Elevated concentration* – *C3‐carnitine* (4.44 mmol/L, RI = 0.14–0.94) – *Total homocysteine* (241 mmol/L, RI < 15) – *Methylmalonic acid* (34.8 mmol/L, RI < 0.45)	Methylmalonic acidemia and homocystinuria, cblC type (#277400)	Based on DNA‐diagnostics
E‐003	B	B	*Elevated* – *Threonine‐N‐acetylgalactosamine* (*z*‐score = 6.5)	*Elevated excretion* – *Alpha‐NAGA biomarkers* (*z*‐scores > 10, RI = *n*.d.)	Alpha‐N‐acetylgalactosaminidase deficiency (#609242)	Based on DNA‐diagnostics
E‐004^b^	B	B	*Elevated* – *3‐Hydroxykynurenine* (*z*‐score = 24.0) – *3‐Methoxykynurenate* (*z*‐score = 8.9) – *Xanthurenic acid* (*z*‐score = 8.5) – *Kynurenine* (*z*‐score = 3.5)	*Elevated excretion* – *Xanthurenic acid* (qualitative, RI = *n.d.*) – *3‐Hydroxykynurenine* (qualitative, RI = *n.d.*)	Kynureninase deficiency (#236800)	Based on DNA‐diagnostics
E‐005	3	3	*Elevated* – *Homocystine* (*z*‐score = 145.5) – *Homocysteine* (*z*‐score = 14.7) – *Cysteinyl‐homocysteine* (*z*‐score = 124.6) – *Methionine* (*z*‐score = 39.7) – *Methionine‐hexose* (*z*‐score = 27.3), – *S‐adenosylmethionine* (*z*‐score = 12.7).	*Elevated concentration* – *Total homocysteine* (305 mmol/L, RI < 15) – *Methionine* (555 mmol/L, RI = 5–43)	Cystathionine beta‐synthase deficiency (#236200)	Based on DNA‐diagnostics
E‐006	B	2	Elevated – *Dihydroorotic acid (z*‐score = 28.3) – *Orotic acid* (*z*‐score = 13.1) – *Uracil* (*z*‐score = 11.7) – *Ureidosuccinic acid* (*z*‐score = 8.8) – *3‐Ureidopropionic acid* (*z*‐score = 5.3) – *Glutamine + Glutamic acid/N‐Methyl‐D‐Aspartic acid* (*z*‐score = 4.0)	*Elevated concentration* – *Glutamine + glutamic acid* (1349 mmol/L, RI = 414–798) *Elevated excretion* – *Orotic acid* (551 mmol/mol creatinine)	Ornithine Carbamoyltransferase deficiency (#311250)	Patient on ICU with hyperammonemia

*Note:* All *z*‐scores were calculated using within‐batch comparison. For metabolomics, *z*‐scores > 2 (elevations) and < −2 (decreases) were considered abnormal. Normal results of disease‐associated metabolites are not included in this table. n.d. = not detectable; RI = reference interval.

^a^ These metabolites were measured after completion of the pilot by an external lab as follow‐up of the results obtained with metabolomics.

^b^ Published in Ref. [15].

The newly detected diagnosis (P‐334) concerned a female patient with a high suspicion of having a urea cycle disorder. Targeted plasma amino acid analysis showed a slight decrease in citrulline, with normal levels of the sum of glutamine and glutamic acid, ornithine, arginine and undetectable argininosuccinate (score 1). A urine sample was not available. The metabolomics profile of this individual showed elevated orotic acid and slightly decreased citrulline (concentrations and *z*‐scores included in Table 2), indicative of ornithine carbamoyltransferase (OTC) deficiency, which was confirmed by genetic analysis that revealed heterozygosity of a pathogenic deletion in the *OTC* gene.

Patient P‐436 was a male patient with intellectual disability and developmental regression for whom targeted analyses in plasma did not show any abnormalities (score = 1). The metabolomics analysis, however, revealed abnormalities of carnitine biosynthesis intermediates, that is, elevation of trimethyllysine (*z*‐score 9.3) and a decrease in deoxycarnitine (gamma‐butyrobetaine; *z*‐score −4.5), which was indicative of epsilon‐trimethyllysine hydroxylase deficiency (TMLHE, OMIM # 300872). These screening results were confirmed by quantitative measurements (see Table 2), but regular genetic testing using short‐read whole genome sequencing did not reveal variants in the *TMLHE* gene. As such, in‐depth genetic analysis is currently being performed with the results still pending.

Finally, six additional IMD diagnoses were made throughout the study period in samples which were excluded from the metabolomics implementation study as they were submitted based on specific genetic findings or as urgent request for diagnostic investigations. In all six analysed samples, aberrant characteristic biomarkers associated with specific IMD were observed (see Table 2), which included the urine marker threonine‐*N*‐acetylgalactosamine in alpha‐*N*‐acetylgalactosaminidase deficiency and different kynurenine metabolites in kynureninase deficiency. Notably, in the sample of the patient with molybdenum cofactor deficiency, many metabolites (> 100) were abnormal, which was related to medication use, poor clinical condition and neonatal age. Nevertheless, a disease‐specific pattern could still be observed, amongst others characterised by decreased levels of nicotinamide degradation products due to deficient aldehyde oxidase activity.

## 4 Discussion

In this metabolomics implementation study, we evaluated the performance of untargeted metabolomics compared with conventional selective metabolic screening for IMD detection in a routine diagnostic setting. Only a few studies have examined prospective screening and implementation in clinical practice until now [9, 10, 11].

Most patients in our study were children and presented with neurocognitive symptoms, in line with the other published cohorts [9, 10, 11] and therefore illustrative of a typical population referred for metabolic screening. The overrepresentation of male patients in our cohort, also previously reported by [10, 11], is probably due to a higher prevalence of the most common indications for metabolic screening (intellectual disability and autism) in males [16].

We found a strong correlation (*r* > 0.7) between metabolite levels measured by targeted assays and metabolomics for most metabolites except serine (borderline: *r* = 0.69) and the sum of glutamine + glutamic acid (*r* = 0.43). We calculated the sum of these metabolites to correct for the well‐known conversion of glutamine into glutamate in stored samples, but this does not factor in non‐enzymatic hydrolysis of glutamate to pyroglutamate [17]. This effect is expected to be minimal or absent when metabolomics is used as a first‐line screening approach as samples undergo shorter storage time and fewer freeze–thaw cycles. Others also assessed correlation for a set of partially overlapping metabolites, revealing equivalent strong correlations, except for free carnitine [4] and several low abundant acylcarnitines [11].

We constructed a scoring system for comparison of the two analysis methods, which focused on interpretation of the metabolite profile in relation to IMD diagnostics and disregarded non‐specific metabolite abnormalities. This approach aligned with the clinical request to screen for IMD. This differs from the scoring system applied by Willems et al. [11], which took into account all metabolite abnormalities, thereby uncovering that the vast majority of samples showed metabolite abnormalities in both targeted and metabolomic analyses. These results stress the necessity of applying a pattern‐recognition approach to IMD diagnostics.

The observed concordance of 85.5% between both approaches indicates that metabolomics largely reproduces the diagnostic information obtained with targeted assays. Similarly, Willems et al. [11] demonstrated that most abnormalities identified by targeted plasma assays were also detected by untargeted metabolomics, specifically amino acids and acylcarnitines. Direct comparison of the concordance rate between the different prospective studies, however, is limited by differences in study population (two different cohorts in Liu et al. [10]) and scoring system [9, 11]. Most discordant results in our study were attributable to metabolites traditionally measured in urine (score B for metabolomics), such as organic acids, that were detected in plasma by metabolomics but not included in targeted plasma analyses. Complementary urinary analyses (targeted or metabolomics‐based) in the same patients may clarify the relevance of these findings in relation to a potential IMD.

Importantly, the frequency of inconclusive results (score 2) requiring follow‐up testing was similar between metabolomics and targeted screening in our study. This finding is clinically relevant, as it suggests that implementation of metabolomics does not increase diagnostic uncertainty or downstream workload. While this specific aspect is less extensively reported in prior studies, it complements the observations by Willems et al. [11] that untargeted metabolomics generates a high number of biochemical deviations that require expert interpretation but do not necessarily translate into clinically meaningful abnormalities.

The additional diagnostic yield of metabolomics in our cohort (yield 0.2% if P‐436 is excluded and 0.4% if included) was modest but clinically meaningful. This relatively low yield is consistent with the findings by Almontashiri et al. [9], who reported a diagnostic yield of 0.7% in their undiagnosed subcohort. In contrast, a substantially higher diagnostic yield has been reported in the study by Liu et al. [10], where untargeted metabolomics achieved a diagnostic rate of 7.1%, approximately sixfold higher than traditional screening. This discrepancy can largely be explained by differences in applied techniques, since they employed four chromatographic analyses (including gas chromatography (GC)–MS) allowing additional measurement of more lipophilic compounds including sterol and lipid biomarkers. Furthermore, they provided a comprehensive and in‐depth analysis integrating all available clinical, genetic and biochemical data in their metabolomics cohort which was compared to isolated biochemical results in their traditional metabolic screening cohort.

Our findings support the clinical value of metabolomics by providing additional diagnostic clues. The detection of a previously unrecognised case of OTC deficiency clearly illustrates the potential of metabolomics to identify disease‐specific biochemical patterns even when complete selective metabolic screening is not possible (e.g., missing urine samples).

Despite these advantages our findings also confirm that untargeted metabolomics cannot fully replace conventional testing. Several IMD identified through urine‐based diagnostics in our cohort were not detected by plasma metabolomics, highlighting a limitation of plasma‐only screening approaches. Comparable shortcomings have been described by Almontashiri et al. [9], who reported missed detection of specific key metabolites such as homogentisic acid. In general, metabolomics has reduced sensitivity for lipophilic compounds and analytes requiring specialised extraction methods (e.g., total homocysteine) and does not detect large molecules such as glycosaminoglycans and proteins (e.g., sialotransferrins). These observations collectively indicate that, at present, metabolomics *in plasma* should be viewed as a complementary approach rather than a complete substitute, particularly for disorders that depend on urine biomarkers or specialised analytical platforms. In this context, the findings of our study are consistent with and extend previous studies by demonstrating that untargeted metabolomics is a robust and clinically valuable tool for IMD screening. In the future, a combined approach integrating *plasma* and *urine* metabolomics (including oligosaccharides and glycosaminoglycan fragments detection), *plasma* lipidomics, total homocysteine and *plasma* CDG screening may be suitable for comprehensive IMD screening.

We experienced that conducting a prospective metabolomics implementation study adds value beyond validation in the process of implementing a novel IMD screening strategy such as metabolomics (see Figure 5). This strategy allowed us to (i) test the diagnostic workflow in real life, (ii) tackle possible technical difficulties and (iii) discuss and harmonise annotation and interpretation amongst technicians and laboratory specialists. Thanks to this approach we managed to have an optimised workflow in place before implementation in the diagnostic setting.

**FIGURE 5:**
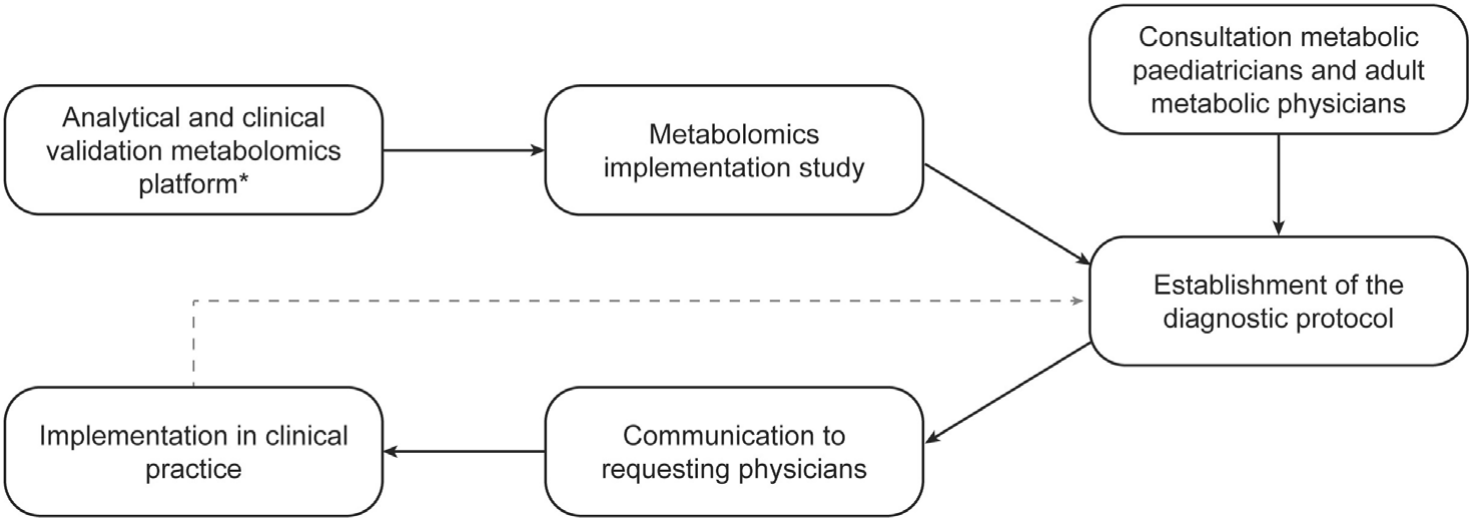
Workflow for development and implementation of our metabolomics platform in clinical practice. The dotted line represents our recommendation to perform periodic evaluation of the protocol and, if considered necessary, adjustment of the protocol. * Published in Ref. [6].

One of these improvements concerned the database used for feature annotation. During the study, a database comprising 879 metabolites was used, including many compounds without a documented association with IMD. This broad annotation scope complicated data interpretation, as aberrant *z*‐scores of non‐disease‐related metabolites frequently obscured clinically relevant abnormalities. To address this limitation, the database was refined into a curated “diagnostic panel” consisting of 322 metabolites with established relevance for IMD (published or reported association with IMD). The implementation of this focused panel not only improved interpretability of metabolomic profiles but also significantly streamlined the workflow by reducing the required time for manual verification of annotated features in a dataset by a technician from approximately 4–3 days. Notably, applying such a predefined diagnostic panel requires periodic updates to incorporate newly discovered biomarkers.

Excluded from this study were emergency requests, follow‐up of positive neonatal screening, therapy monitoring and functional characterisation of VUSs. Practical considerations were that emergencies, neonatal screening follow‐up and (to a lesser extent) therapy monitoring require prompt, specific quantitative results—all which our current metabolomics platform precludes. From an ethical point of view, we averted unwanted incidental findings in these cases. Lastly, we aimed the pilot to represent our [intended] patient population as much as possible; including VUS‐follow‐up would have interfered the prospective nature of the study. Prior to implementation of metabolomics in clinical practice, we involved representatives from both paediatric and adult metabolic medicine to incorporate their clinical perspectives into the workflow design. Together, we identified specific (additional) patient groups for whom metabolomics screening may be less suitable, either for clinical reasons—such as acute and severe disease states (e.g., ICU patients) where numerous secondary abnormalities may obscure interpretation—or for logistical considerations, including the need for rapid turnaround times in neonatal cases. For these patient groups and clinical scenarios, we continue to perform targeted diagnostic testing. Accordingly, our current implementation is best regarded as a metabolomics‐based screening approach for non‐urgent clinical presentations. The scope of implementation may vary between laboratories, depending on the turnaround time achievable for metabolomics analyses relative to targeted diagnostic tests.

In conclusion, our results from this metabolomics implementation study support the implementation of untargeted metabolomics for clinical screening of IMD, in conjunction with complementary approaches, according to the workflow depicted in Figure 5. Following implementation, periodic evaluation is recommended to continuously monitor and improve performance in clinical practice. We anticipate that sharing our detailed protocol and implementation strategy will facilitate and encourage colleagues in the IMD field to adopt an untargeted metabolomics screening approach in their laboratories as well.

## Author Contributions

D. Augustijn, M.C. Hagemeijer, J. Bierau, E.H. Jacobs, S.M.I. Goorden and G.J.G. Ruijter conceptualised and designed the study and drafted the manuscript. R. Bonte, C.J. Klein‐de Ronde, E. van der Star and M. van Dijk collected and analysed data. D.V. contributed to bioinformatics analyses and data infrastructure. D.Y. van Haaften‐Visser, N.C. Peltenburg‐de Jong and C. Debuy critically reviewed and revised the manuscript. All authors approved the final manuscript as submitted and agreed to be accountable for all aspects of the work.

## Funding

The authors have nothing to report.

## Ethics Statement

This article does not contain any studies with animal subjects.

## Conflicts of Interest

The authors declare no conflicts of interest.

## Supporting information


**Supplementary Figure 1.** Most frequently observed symptoms stratified by sex. The asterisk indicates a significant difference between sex based on a Chi‐square test. The Chi‐square test was performed when ≥ 10 patients presented with the symptom.
**Supplementary Figure 2**. Correlation between concentrations of metabolites in targeted tests and *z*‐scores in metabolomics. The reference intervals covering all ages for the metabolites are depicted in grey.
**Supplementary Figure 3**. An example *z*‐score plot (elevations of metabolites in negative mode are depicted) of patient E‐005 (see Table 2 in main text). Crosses depict within‐batch *z*‐scores (comparison with other samples in the same batch); circles between‐batch *z*‐scores (comparison with samples from at least 7 previous batches) and triangles between‐batch *z*‐scores matched for age and sex (using regression). Measurements were performed in triplicate.
**Supplementary Table 1**. Table outlining samples with discrepant scores.

## Data Availability

The data that support the findings of this study are available on request from the corresponding author.
