# Supplementary material for: Bringing Untargeted Metabolomics to the Clinic: Real‐World Data and Implementation for Screening of Inherited Metabolic Diseases
Source: J Inherit Metab Dis. 2026 Nov 9;49(6):e70260. doi: 10.1002/jimd.70260 (PMC13748796; doi:10.1002/jimd.70260)
Supplement: Supplementary file 1 [file JIMD-49-0-s001.docx]

**Supplemental Information**

**Supplemental Figure 1:** **Most frequently observed symptoms stratified by sex.** The asterisk indicates a significant difference between sex based on a Chi-square test. The Chi-square test was performed when ≥ 10 patients presented with the symptom.

**Supplemental Figure 2:** **Correlation between concentrations of metabolites in targeted tests and z-scores in metabolomics**. The reference intervals covering all ages for the metabolites are depicted in grey.

**Supplemental Figure 3**: An example z-score plot (elevations of metabolites in negative mode are depicted) of patient E-005 (see Table 2 in main text). Crosses depict within-batch z-scores (comparison with other samples in the same batch); circles between-batch z-scores (comparison with samples from at least 7 previous batches) and triangles between-batch z-scores matched for age and sex (using regression). Measurements were performed in triplicate.

**Supplemental Table 1:** **Table outlining samples with discrepant scores.**

| **Sample** | **Urine received?** | **Score metabolomics** | **Score Targeted test** | **Group of metabolites** | **Aberrant metabolites metabolomics** | **Aberrant metabolites targeted tests** |
| --- | --- | --- | --- | --- | --- | --- |
| P-009 | Yes | 2 | 1 | Amino acids | Elevated arginine (z-score = 2.4), arginine-hexose (z-score = 2.3) and homoarginine (z-score = 1.5). Determine amino acids as next step. | Arginine is slightly elevated (139 μmol/L, RI: 32 – 128). |
| P-015 | Yes | 2 | 1 | Acylcarnitines & organic acids | Elevated glutarylcarnitine (C5DC, z-score = 7.9) and ethylmalonic acid (z-score = 6.1). Determine acylcarnitines and organic acids in urine. | C5DC-carnitine is normal (0.04 μmol/L, RI = 0 – 0.06). The excretion of ethylmalonic acid is normal in urine (4.2 mmol/mol creatinine, RI: 0 – 20). |
| P-018 | Yes | 2 | 1 | Acylcarnitines & organic acids | Elevated ethylmalonic acid (z-score = 10.3) and butyrylcarnitine (C4) (z-score = 7.1). Determine acylcarnitines and organic acids in urine. | C4-carnitine is normal (0.51 μmol/L, RI = 0.07 – 0.58). The excretion of ethylmalonic acid is normal in urine (3.0 mmol/mol creatinine, RI: 0 – 20). |
| P-022 | Yes | 1 | 2 | Amino acids | Glutamine + glutamic acid / N-methyl-D-aspartic acid is normal (z-score =0.2). | The sum of glutamine and glutamic acid is elevated (828 μmol/L, RI = 414 – 798). Suggested to determine ammonia. |
| P-024 | Yes | 2 | 1 | Amino acids | Elevated N-acetylglycine (z-score = 8.5) and glycine-hexose (z-score = 2.9). Determine amino acids as next step. | Glycine is normal (290 μmol/L, RI = 97 – 389). |
| P-029 | No | 1 | 2 | Amino acids | Alanine / β-alanine / sarcosine is slightly elevated (z-score = 2.2). | Elevated alanine (709 μmol/L, RI = 176 – 480). Suggested to determine lactic acid in blood. |
| P-033 | Yes | 1 | 2 | Amino acids | Proline is slightly elevated (z-score = 2.9) and alanine / β-alanine / sarcosine are normal (z-score = 1.2). | Elevated alanine (757 μmol/L, RI = 176 – 480) and proline (630 μmol/L, RI = 89 – 281). Suggested to determine lactic acid in blood. |
| P-057 | No | 2 | 1 | Acylcarnitines | Elevated isobutyrylcarnitine (C4) (z-score = 7.0). Determine acylcarnitines as next step. | C4-carnitine is slightly elevated (0.71 μmol/L, RI = 0.07 – 0.58 μmol/L). |
| P-066 | Yes | B | 1 | Organic acids | Elevated mevalonic acid in metabolomics (z-score = 2.1). | Mevalonic acid is normal in urine (not detected). |
| P-077 | Yes | B | 1 | Organic acids | Elevated mevalonic acid in metabolomics (z-score = 4.9). | Mevalonic acid is normal in urine (not detected). |
| P-093 | Yes | 1 | B | Organic acids | Normal metabolomics profile. Both targeted metabolites not found in metabolomics. | Elevated excretion of tiglylglycine (9 mmol/mol creatinine, RI = 0 – 5) and excretion of 2-methyl-3-hydroxybutyric acid. Diagnosis: 2-methyl-3-hydroxybutyryl-CoA dehydrogenase (MHBD, HSD17B10) deficiency. Confirmed by DNA-diagnostics. |
| P-102 | Yes | 1 | 2 | Amino acids | Proline is slightly elevated (z-score = 2.3) and alanine / β-alanine / sarcosine are normal (z-score = 0.8). | Elevated alanine (577 μmol/L, RI = 176 – 480) and proline (393 μmol/L, RI = 89 – 281). Suggested to determine lactic acid in blood. |
| P-110 | Yes | 1 | 2 | Amino acids | Elevated methionine + methioninesulfoxide (z-score = 6.0) and methionine (z-score = 5.3). | Elevated methionine (90 μmol/L, RI = 18 – 42). Suggested to measure homocysteine. |
| P-127 | Yes | 1 | 2 | Acylcarnitines | Normal propionylcarntine (C3, z-score = 1.0) and methylmalonic acid (z-score = 0.3). | C3-carnitine is slightly elevated (1.48 μmol/L, RI = 0.14 – 0.94). Next step to determine methylmalonic acid (0.35 μmol/L, RI < 0.45). |
| P-141 | Yes | B | 1 | Oligosaccharides | Elevated sialic acid (z-score = 5.6). | The excretion of sialic acid in urine is normal (53 mmol/mol creatinine, RI = 0 – 97). |
| P-146 | Yes | B | 1 | Organic acids | Elevation of 4-hydroxybutyric acid (z-score = 5.8). | The excretion of 4-hydroxybutyric acid is normal in urine (0.9 mmol/mol creatinine, RI = 0 – 2). |
| P-155 | Yes | B | 1 | Organic acids | Elevation of N-acetyl-S-(2-carboxypropyl)cysteine (z-score = 9.0) and S-(2-carboxypropyl)cysteine (z-score = 8.6). No 2-methyl-2,3-dihydroxybutyric detected. | 2-methyl-2,3-dihydroxybutyric acid is not detected in urine. |
| P-158 | Yes | B | 2 | Purines and pyrimidines | Elevations of different purines and pyrimidines (e.g. Air, dihydrothymine, xanthine) | Purines and pyrimidines are normal (does not include all purines/pyrimidines as measured in metabolomics). |
| P-163 | Yes | A | 1 | Specific test | Elevated pipecolic acid in metabolomics (z-score = 13.0). | Pipecolic acid in plasma is elevated (26941 nmol/L, ref. interval: 694 – 5324). |
| P-166 | No | 2 | 1 | Acylcarnitines & organic acids | Elevated orotic acid (z-score = 9.3) and C5OH-carnitine (z-score = 6.1). Determine amino acids, acylcarnitines and organic acids in urine. | C5OH-carnitine is slightly elevated (0.26 μmol/L, RI = 0.07 – 0.22). Normal amino acid profile. No urine sample received for analysis. |
| P-173 | Yes | B | 1 | Organic acids | Elevated mevalonic acid in metabolomics (z-score = 3.0). | Mevalonic acid is normal in urine (not detected). |
| P-210 | Yes | A | 1 | Specific test | Elevated pipecolic acid in metabolomics (z-score = 8.7). | Pipecolic acid in plasma is elevated (23458 nmol/L, ref. interval: 694 – 5324). |
| P-212 | Yes | 1 | B | Acylcarnitines & organic acids | Propionylcarnitine (C3, z-score = 1.0) is normal. MMA is not detected. | Elevated excretion of methylmalonic acid (107 mmol/mol creatinine, RI = 0 – 25).  Add methylmalonic acid in plasma (elevated 6.52 μmol/L, RI = 0 – 0.45) and acylcarnitines (C3-carnitine is elevated, 1.28 μmol/L, RI = 0.14 – 0.94). normalised later in life. |
| P-213 | Yes | 2 | 1 | Amino acids | Elevated homocysteine (z-score = 36.0) and methionine-hexose (z-score = 7.9). Determine amino acids and homocysteine as next step. | Elevated homocysteine (22.9 μmol/L, RI < 15) and normal concentration methionine (27 μmol/L, RI = 18 – 42). |
| P-216 | No | B | 1 | Organic acids | Elevated mevalonic acid in metabolomics (z-score = 4.3). | No urine sample received for analysis. |
| P-218 | Yes | 1 | 2 | Acylcarnitines | Slightly elevated octanoylcarnitine (C8, z-score = 4.2) and decanoylcarnitine (C10, z-score = 3.6). | Elevated C8-carnitine (0.40 μmol/L, RI = 0.04 – 0.22) and C10-carnitine (0.5 μmol/L, RI = 0 – 0.3). Elevated C8/C10 ratio (0.062, RI = 0 – 0.049). Suggested to measure riboflavin (vitamin B2). |
| P-222 | Yes | 2 | 1 | Acylcarnitines | Elevated glutarylcarnitine (C5DC, z-score = 6.0). Determine acylcarnitines as next step. | Slightly elevated C5DC-carnitine (0.07 μmol/L, RI = 0 – 0.06). |
| P-232 | Yes | A | 1 | Specific test | Elevated pipecolic acid in metabolomics (z-score = 11.6). | Pipecolic acid in plasma is elevated (7885 nmol/L, ref. interval: 694 – 5324). |
| P-235 | Yes | 2 | 1 | Amino acids | Elevated glycine (z-score = 2.6) and glycine-hexose (z-score = 2.6). Determine amino acids as next step. | Normal concentration of glycine (386 μmol/L, RI = 112 – 424). |
| P-238 | No | B | 1 | Several | Many metabolites elevated. No specific pattern. Targeted tests as next step. | Elevation of various amino acids and acylcarnitines. |
| P-253 | Yes | B | 1 | Organic acids | Elevated N-acetylaspartic acid (z-score = 4.9) and N-acetylaspartylglutamic acid (z-score = 2.0). | The excretion of N-acetylaspartic acid is normal (13.1 mmol/mol creatinine, RI = 0 – 40). |
| P-257 | Yes | B | 1 | Oligosaccharides | Elevated sialic acid (z-score = 4.1). | The excretion of sialic acid in urine is normal (4.4 mmol/mol creatinine, RI = 0 – 12). |
| P-258 | Yes | 1 | B | Organic acids | Methylcitric acid is normal (z-score = -1.2) in plasma. MMA is not detected. | Elevated excretion of methylmalonic acid (25 mmol/mol creatinine, RI = 0 – 20) and methylcitric acid (12 mmol/mol creatinine, RI = 0 – 5). |
| P-264 | No | B | 2 | Purines and pyrimidines & acylcarnitines | Elevations of different purines and pyrimidines (xanthosine, hypoxanthine, xanthine). No elevations of acylcarnitines. | Multiple acylcarnitines are slightly elevated. Advice to measure riboflavin (vitamin B2). No urine sample to measure purines and pyrimidines. |
| P-272 | Yes | B | 2 | Several | Many metabolites elevated. No specific pattern. Targeted test as next step. | Several metabolites are elevated. Decision to repeat measurements left to clinical indication. |
| P-275 | No | 1 | 2 | Amino acids | Elevated glycine (z-score = 6.1) and glycine-hexose (z-score = 2.6). | Elevated glycine (517 μmol/L, RI = 112 – 424). New plasma sample requested to repeat glycine measurement. |
| P-282 | Yes | 1 | B | Organic acids | Elevated suberic acid (z-score = 5.4). Adipic acid is not detected. | Elevated excretion of adipic acid (174 mmol/mol creatinine, RI = 0 – 25) and suberic acid (47 mmol/mol creatinine, RI = 0 – 15). |
| P-307 | Yes | B | 1 | Bile acids | Elevated cholestanepentol + gluc (1) (z-score = 8.6) and cholestanepentol + gluc (4) (z-score = 2.9)* | The excretion of cholestanepentol + glucuronidine is normal (0.01 mmol/mol creatinine, RI = 0 – 0.038) |
| P-296 | No | 2 | 1 | Acylcarnitines | Elevated 3-hydroxyisobutyrylcarnitine (C4OH, z-score = 7.6). Determine acylcarnitines as next step. | Normal C4OH-carnitine (0.13 μmol/L, RI = 0.0 – 0.15). |
| P-320 | No | 2 | 1 | Acylcarnitines | Elevated 3-hydroxyisobutyrylcarnitine (C4OH, z-score = 12.8). Determine acylcarnitines as next step. | Slightly elevated C4OH-carnitine (0.17 μmol/L, RI = 0.0 – 0.15). |
| P-325 | Yes | B | 1 | Organic acids | Elevated mevalonic acid in metabolomics (z-score = 5.2). | Mevalonic acid is normal in urine (not detected). |
| P-331 | Yes | 2 | 1 | Acylcarnitines | Elevated decanoylcarnitine (C10, z-score = 2.9), dodecanoylcarnitine (C12, z-score = 3.5) and 5-tetradecenoylcarnitine (C14:1, z-score = 3.6). Determine acylcarnitines as next step. | Normal concentration of C10-carnitine (0.3 μmol/L, RI = 0.0 – 0.3), C12-carnitine (0.14 μmol/L, RI = 0.0 – 0.14) and C14:1 (0.18 μmol/L, RI = 0.02 = 0.18). |
| P-334 | No | B | 1 | Organic acids & amino acids | Elevated orotic acid (z-score = 9.0) and slightly decreased citrulline (z-score = -2.4). Suspected of urea cycle defect. | Slightly decreased citrulline (7.7 μmol/L, RI = 16 – 56). No urine sample received for analysis.  Diagnosis of OTC confirmed by DNA diagnosis. |
| P-341 | Yes | 1 | B | Organic acids | No MMA detected in metabolomics. Methylcitric acid is slightly elevated (z-score = 2.7). | Elevated excretion of methylmalonic acid (53 mmol/mol creatinine, RI = 0 – 25) and methylcitric acid (15 mmol/mol creatinine, RI = 0 – 5) in urine. Measured acylcarnitines in urine which were normal. |
| P-351 | Yes | 1 | B | Mucopolysaccharides | Normal metabolomics profile. | Elevated excretion of total mucopolysacharides (8.2 mmol/mol creatinine, RI = 0 - 6.7) and heparan sulphate (6.9 mmol/mol creatinine, RI = 0 – 1.8). MPSIII3B diagnosis based on DNA diagnostics and reduced enzyme activity of α-N-acetylglucosaminidase. |
| P-357 | No | B | 1 | Sugars and sugar alcohols | Elevated mannitol/sorbitol/galactitol (z-score = 25.0), seduheptulose/mannoheptulose (z-score = 15.6) and arabitol/ribitol/xylitol (z-score = 3.1). | No urine sample received for analysis of sugars and sugar alcohols. |
| P-359 | Yes | 2 | 1 | Amino acids | Decreased serine (z-score = -2.6). Determine amino acids as next step. | Normal concentration of serine (126 μmol/L, RI = 88 – 288). |
| P-363 | Yes | B | 1 | Purines and pyrimidines | Elevated 2’-deoxyadenosine (z-score = 6.6). | 2’-Deoxyadenosine is not detected in urine. |
| P-371 | Yes | 2 | 1 | Acylcarnitines | Elevated 5-tetradecenoylcarnitine (C14:1, z-score = 5.6) and 5,8-tetradecadienoylcartinine (C14:2, z-score = 3.4). Determine acylcarnitines as next step. | Normal C14:1-carnitine (0.13 μmol/L, RI = 0.02 – 0.18) and slightly elevated C14:2-carnitine (0.09 μmol/L, RI = 0.00 – 0.08). |
| P-373 | No | B | 1 | Organic acids | Elevated ethylmalonic acid (z-score = 5.9) in metabolomics. | No urine sample received for analysis. Targeted analysis of C4-carnitine is normal (0.45 mmol/L, RI: 0.07 – 0.58). |
| P-374 | Yes | B | 2 | Organic acids, acylcarnitines & amino acids | Elevated 2-methylbutyrylglycine (z-score = 8.8) and tiglylcarnitine (C5:1, z-score = 4.4).  Alanine / β-alanine / sarcosine (z-score = 1.2) and proline (z-score = 1.1) are normal. | There is no 2-methylbutyrylglycine detected in the urine. C5:1-carnitine in plasma is normal (0.01 μmol/L, RI = 0 – 0.04).  Elevated alanine (520 μmol/L, RI = 176 – 480) and proline (314 μmol/L, RI = 89 – 281). Suggested to determine lactic acid in blood. |
| P-384 | Yes | 1 | 2 | Amino acids | Alanine / β-alanine / sarcosine are normal (z-score = 0.7). | Elevated alanine (533 μmol/L, RI = 220 – 480). Suggested to determine lactic acid in blood. |
| P-386 | Yes | B | 1 | Purines and pyrimidines | Elevated orotic acid (z-score = 5.4) and ureidosuccinic acid (z-score = 5.1). | The excretion of orotic acid is normal (2.7 mmol/mol creatinine, RI = 0 – 3) |
| P-391 | Yes | 2 | 1 | Several | Many metabolites elevated. No specific pattern. Targeted tests as next step. | Slightly elevation of various acylcarnitines. |
| P-392 | Yes | B | 1 | Purines and pyrimidines | Elevated Succinyladenosine (z-score = 3.9) and SAICAr (z-score = 3.6). | Succinyladenosine and SAICAr are not detected in urine. |
| P-404 | Yes | B | 1 | Purines and pyrimidines | Elevated 2'-Deoxyadenosine (z-score = 7.2). | Deoxyadenosine is not detected in urine. |
| P-426 | Yes | 1 | 2 | Amino acids | Alanine / β-alanine / sarcosine are normal (z-score = 0.7). | Elevated alanine (569 μmol/L, RI = 220 – 480). Suggested to determine lactic acid in blood. |
| P-427 | Yes | 1 | 2 | Amino acids | Alanine / β-alanine / sarcosine (z-score = 0.5) and proline (z-score = 0.2) are normal. Glutamine + glutamic acid / N-methyl-D-aspartic acid is normal (z-score = -0.4) | Elevated alanine (612 μmol/L, RI = 176 – 480) and proline (334 μmol/L, RI = 89 – 281). Suggested to determine lactic acid in blood. Glutamine is elevated (815 μmol/L, RI = 400 – 720). Suggested to determine ammonia. |
| P-431 | No | B | 1 | Purines and pyrimidines | Elevated xanthosine (z-score = 6.7), hypoxanthine (z-score = 6.5) and xanthine (z-score = 2.6). | No urine sample received for analysis of purines and pyrimidines. |
| P-433 | Yes | B | 1 | Organic acids | Elevated 3-methylglutaric acid (z-score = 13.8). | The excretion of 3-methylglutaric acid is normal (1.4 mmol/mol creatinine, RI = 0 – 5). |
| P-435 | Yes | 1 | 2 | Organic acids | Methionine is normal (z-score = -1.7). | Decreased methionine (14 μmol/L, RI = 18 – 42). Add homocysteine as extra test which was normal (11.9 μmol/L, RI < 15). |
| P-436 | No | A | 1 | Carnitine biosynthesis intermediates | Elevated trimethyllysine (z-score = 9.3) and decreased deoxycarnitine (z-score = -4.5). Indicative of TMLHE deficiency. | Elevated N-6-trimethyllysine (2.47 μmol/L, RI = 0.2 – 1.2) and decreased deoxycarnitine (0.06 μmol/L, RI = 0.3 – 1.4). Indicative of TLMHE deficiency. |
| P-439 | Yes | B | 1 | Purines and pyrimidines | Various nucleosides are elevated. No specific pattern. | Elevated excretion of uridine (21.5 mmol/mol creatinine, normally not detected). Other purines and pyrimidines in urine is normal. |
| P-442 | Yes | 1 | B | Mucopolysaccharides | Normal metabolomics profile. | Elevated excretion of total mucopolysacharides (17.4 mmol/mol creatinine, RI = 0 – 11.2), heparan sulphate (18.3 mmol/mol creatinine, RI = 0 – 1.8), dermatan sulphate (10.6 mmo/mmol creatinine, RI = 0 – 3.0). Diagnosis of MPSI confirmed by DNA diagnosis of the IDUA gene and reduced enzyme activity of α-L-iduronidase. |
| P-447 | Yes | B | 1 | Oligosaccharides | Elevated thr-galnac (z-score = 5.3). | No indications for NAGA deficiency in oligosaccharides analysis. |
| P-454 | Yes | B | 1 | Purines and pyrimidines | Elevated AICAr (z-score 4.2). | No AICAR excretion in urine. |
| P-456 | Yes | B | 1 | Several | Many metabolites elevated, mostly measured targeted in urine. No specific pattern. Targeted tests as next step. | Both plasma and urine give no indication for an inborn error of metabolism. |
| P-459 | No | B | 1 | Organic acids | Elevated lactic acid (z-score = 4.3). Suggested to measure lactic acid in plasma. | Normal alanine (479 μmol/L, RI = 176 – 480) and proline (177 μmol/L, RI = 89 – 281) in plasma. |
| P-469 | No | 2 | 1 | Several | Many metabolites elevated. No specific pattern. Targeted tests as next step. | Elevation of various amino acids and acylcarnitines. |
| P-474 | Yes | 1 | B | Amino acids | Normal metabolomics profile. | Elevated excretion of phosphoethanolamine in urine (258 mmol/mol creatinine, RI = 0 – 40). Hypophosphatasia confirmed by DNA diagnostics of the ALPL gene. |

Samples in grey are IMD diagnoses (see Table 2). All z-scores were calculated using within-batch comparison. Isomers that could not be distinguished are indicated with a forward slash (‘’/’’) (for example Alanine / β-alanine / sarcosine). Pipecolic acid, N-6-trimethyllysine and deoxycarnitine (=gamma-butyrobetaine) were quantitatively measured when found to be elevated in the metabolomics screening results. RI=reference interval.

* Cholestanepentol + gluc (1) and cholestanepentol + gluc (4): indicate different glucuronic acid derivates which are formed from cholestanepentol (established biomarker for CTX). These could be distinguished based on retention time, but the lack of authentic standards precluded identification. Therefore, we arbitrarily named them by adding an increasing number in line with increasing retention time.
